# Measuring dengue illness intensity: Development and content validity of the dengue virus daily diary (DENV-DD)

**DOI:** 10.1186/s41687-023-00624-5

**Published:** 2023-08-23

**Authors:** Amy M. Jones, Todd L. Saretsky, Charlotte Panter, Jane R. Wells, Frances White, Verity Smith, Helen Kendal, Kevin Russell, Madelyn Ruggieri, Shawna R. Calhoun, Adam Gater, Justin O’Hagan, Kathryn B. Anderson, Valerie A. Paz-Soldan, Amy C. Morrison, Lisa Ware, Michelle Klick, Stephen Thomas, Morgan A. Marks

**Affiliations:** 1 Adelphi Values Ltd., Patient Centered Outcomes, Cheshire, UK; 2 Merck & Co., Inc., Rahway, NJ USA; 3 Sanofi, Reading, Berkshire UK; 4 Institute for Global Health and Translational Sciences, State University of New York, Upstate Medical University, Syracuse, NY USA; 5 Department of Tropical Medicine, Tulane School of Public Health and Tropical Medicine, New Orleans, LA USA; 6 Department of Pathology, Microbiology, and Immunology, School of Veterinary Medicine, University of California, Davis, CA USA; 7 Moderna, Cambridge, MA USA

**Keywords:** Clinical outcome assessment (COA), Cognitive debriefing, Concept elicitation, Dengue, Dengue human infection model (DHIM), Observer-reported outcome (ObsRO), Patient-reported outcome (PRO), Qualitative, Quantitative

## Abstract

**Background:**

Dengue is the most prevalent arboviral infection causing an estimated 50–60 million cases of febrile illness globally per year, exacting considerable disease burden. Few instruments exist to assess the patient illness experience, with most based on healthcare provider assessment, lacking standardization in timepoints and symptom assessment. This study aimed to evaluate the content validity of the novel ‘Dengue Virus Daily Diary (DENV-DD)’, designed to measure symptom intensity and disease burden within outpatient infant to adult populations.

**Methods:**

The Dengue Illness Index Report Card was used as a foundation to create the DENV-DD, consisting of patient- and observer-reported outcome (PRO/ObsRO) instruments. In two South American dengue-endemic communities, qualitative combined concept elicitation and cognitive debriefing interviews were conducted among individuals and caregivers of children with symptomatic laboratory-confirmed dengue. Interviews were conducted across two rounds allowing DENV-DD modifications. A small-scale quantitative assessment of the DENV-DD was also conducted with data from an independent Dengue Human Infection Model (DHIM) to generate early evidence of feasibility of DENV-DD completion, instrument performance and insight into the sign/symptom trajectory over the course of illness.

**Results:**

Forty-eight participants were interviewed (20 adults, 20 older children/adolescents with their caregivers, 8 caregivers of younger children). A wide spectrum of signs/symptoms lasting 3–15 days were reported with fever, headache, body ache/pain, loss of appetite, and body weakness each reported by > 70% participants. DENV-DD instructions, items and response scales were understood, and items were considered relevant across ages. DHIM data supported feasibility of DENV-DD completion.

**Conclusions:**

Findings demonstrate content validity of the DENV-DD (PRO/ObsRO instruments) in dengue-endemic populations. Psychometric and cultural validity studies are ongoing to support use of the DENV-DD in clinical studies.

**Supplementary Information:**

The online version contains supplementary material available at 10.1186/s41687-023-00624-5.

## Abstract (plain language summary)

Dengue is the most common viral infection transmitted to humans by mosquitos, and affects an estimated 50–60 million individuals globally per year. However, there are few resources for understanding and capturing the patient experience of dengue throughout illness. Most research studies are based on healthcare provider assessment, which lack consistency in terms of assessment time points and the signs/symptoms assessed. The ‘Dengue Illness Index Report Card (DII-RC)’ was used as a foundation to create the new ‘Dengue Virus Daily Diary (DENV-DD)’ to better capture the patient experience of symptom intensity and dengue disease burden for the duration of illness. Forty-eight individuals and caregivers of younger children from Peru and Ecuador who recently had symptomatic dengue were interviewed to understand the patient experience over the time of illness and to test whether the DENV-DD is understood by patients and caregivers and includes all relevant and important signs/symptoms and health-related quality of life impacts. Nine individuals with active dengue infection also completed the DENV-DD daily for 28-days as part of a clinical study. We found that > 70% of patients experienced fever, headache, body ache/pain, loss of appetite and body weakness. The DENV-DD instructions, questions and response option(s) were well understood, feasible to complete and the concepts assessed by the DENV-DD were relevant to the dengue experience. Our study adds to the understanding of the dengue illness experience and supports the DENV-DD for use in future dengue studies as an assessment of signs/symptoms throughout the duration of illness.

**Supplementary Information:**

The online version contains supplementary material available at 10.1186/s41687-023-00624-5.

## Background

Dengue is a mosquito-borne viral illness endemic in more than 100 countries with cyclical epidemics in the Americas, South-East Asia, and Western Pacific regions [1]. In 2019, the World Health Organization (WHO) reported 5.2 million cases of symptomatic dengue globally based on country and regional level passive surveillance systems [2]. However, it is recognized that dengue prevalence is significantly under-reported using passive surveillance methods. The actual number of symptomatic cases is estimated to affect 50–60 million individuals globally per year [3–6].

Field studies and human infection models [7–9] typically characterize dengue illness by fever, headache, musculoskeletal pain, fatigue, rash and nausea/vomiting, in line with the WHO definition [10]. There is no specific antiviral treatment for dengue illness and most treatments aim to only alleviate symptoms. Most signs/symptoms of dengue typically resolve within 5–14 days after onset, with the majority of cases being treated in outpatient settings. However, clinically severe illness can develop resulting in hypotension and organ dysfunction from plasma leakage and/or internal bleeding. Failure to manage fluid replacement can lead to shock, multi-organ failure, fluid overload and death [3].

Despite its prevalence, few resources exist for understanding and capturing the patient experience of dengue (signs/symptoms and impacts on health-related quality of life [HRQoL]) throughout the course of illness, i.e. disease burden. Most existing instruments are based on healthcare provider assessment, which can be highly variable between providers, lacking standardization in timepoints and symptom assessment [11, 12]. Standardized patient-completed instruments are needed to adequately characterize disease burden.

Recent surveys have sought to establish detailed information regarding symptoms, including intensity and duration. However, a more concise measure prioritizing concepts important to patients is needed for use in large scale or multinational clinical and cohort studies to alleviate completion burden. The Dengue Illness Index Report Card (DII-RC), a 16-item daily diary developed to assess patients’ or caregivers' subjective experience of dengue illness, was identified. However, the DII-RC captures only the presence or absence of commonly observed signs/symptoms (versus illness intensity). The developers acknowledged further adaption should be undertaken to optimize use in clinical studies by following regulatory guidance for clinical outcome assessment (COA) development [13]. Therefore, using the DII-RC as a foundation, a new COA instrument (the Dengue Virus Daily Diary [DENV-DD], consisting of patient- and observer-reported outcome (PRO/ObsRO) instruments) was developed, in line with regulatory guidance [14–16] to evaluate the trajectory and intensity of dengue-associated signs/symptoms over the course of a patient’s illness, from the patient or caregiver perspective.

This study aimed to refine and evaluate the content validity and feasibility of the DENV-DD (PRO and ObsRO instruments) as a first step in its validation to assess dengue illness symptom intensity and burden in infants to adults, for clinical research and real-world use.

## Methods

**Fig. 1:**
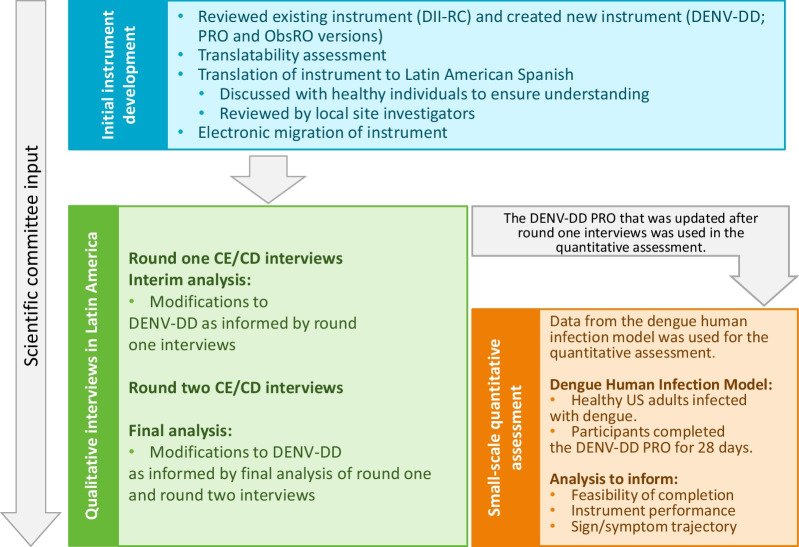
Study overview. *DII-RC* dengue illness index report card, *CE* concept elicitation, *CD* cognitive debriefing, *PRO* patient reported outcome, *ObsRO* observer reported outcome, *DENV-DD* dengue virus daily diary

An overview of the study design is summarized in Fig. 1. At key stages throughout the research, a scientific committee composed of clinical experts in dengue (AL, BC, KR, ST, TE, [See acknowledgements]) provided input and guidance.

### Initial instrument development

**Fig. 2:**
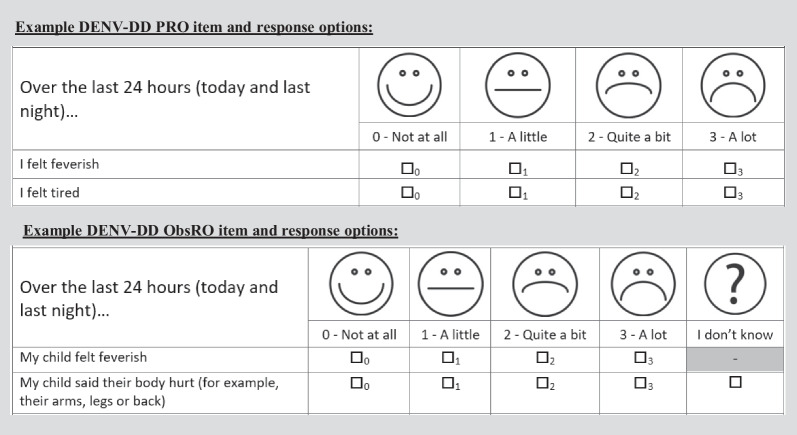
Example DENV-DD PRO and ObsRO item and response options

The DII-RC underwent a face-validity assessment by COA development experts, including a review of WHO dengue guidelines [10] to form the draft DENV-DD. An item-refinement meeting was held with the scientific committee to gain consensus on instruction/item wording. Two US English versions were developed, with age bands guided by International Society for Pharmacoeconomics and Outcomes Research recommendations [17]: a PRO for self-completion by adults (aged 18+ years) and older children/adolescents (aged 8–17 years) with a caregiver present (if needed); and an ObsRO for completion by caregivers of young children (aged 1–7 years) with symptomatic dengue. Key differences included: the PRO contained additional pain-related items (e.g. muscle and bone) which caregivers are unlikely to be able to report on; the ObsRO contained items assessing ‘sleeping more’ and ‘feeling grumpy’ as observable indicators of fatigue and general illness in children as well as an ‘I don’t know’ response option for items that cannot be directly observed in very young children. While the DII-RC only captured presence or absence of symptoms via a dichotomous response scale, for the DENV-DD, the response scale was updated to include multiple response options (Fig. 2). These are defined according to verbal descriptors and pictorial faces and designed to capture gradation in symptom intensity and facilitate evaluation of changes in symptom intensity over time (to be assessed during later planned psychometric validation work, in line with best practice guidance [14]). Two global items, assessing dengue illness intensity and daily impact of dengue illness respectively, were also adapted from the DII-RC to capture illness burden on daily life and an item to input the patient’s temperature (assessed by self/caregiver administration using a thermometer) added. Items were reworded to ensure use of patient-friendly language and a 24-h recall period was employed. This resulted in a draft 22-item PRO containing 19 symptom items, 2 global items, and 1 temperature item, and a 21-item ObsRO containing 18 symptom items, 2 global items, and 1 temperature item.

Both versions were subjected to a translatability assessment [18] (assessing Thai, Malay, Indonesian, Vietnamese, Filipino/Tagalog, Traditional Chinese, Korean, Japanese) to confirm the appropriateness of the wording; and the suitability of concepts for future translations in countries where dengue is endemic [19]. The DENV-DD underwent forward–backward translation into Spanish, using country-neutral words but ensuring cultural relevance for the sample in Peru and Ecuador; and was migrated onto an electronic platform for the interviews. In accordance with best practice for linguistic validation, the draft DENV-DD was discussed with healthy individuals in Iquitos, Peru (prior to qualitative interviews) to ensure terms and concepts aligned with the local idioms and general reading level.

### Qualitative interviews

A non-interventional, cross-sectional, qualitative study was conducted. This involved interviews in two dengue endemic regions (Iquitos, Peru and Machala, Ecuador) with individuals and caregivers of younger children who recently experienced laboratory-confirmed dengue. The regions were selected as they are recognized as key areas of dengue research [2, 20–24]. The interviews comprised combined concept elicitation (CE) and cognitive debriefing (CD) activities: CE explored the patient experience of dengue and informed development of a conceptual model and assessment of the conceptual comprehensiveness of the DENV-DD, and CD assessed whether the DENV-DD is understood, relevant and captures all concepts important to patients. Interviews were conducted in two rounds, allowing for modifications and testing of the updated instrument between rounds. All participant-facing study documents and interview guides were translated by certified translators and reviewed by local site investigators to ensure they were culturally sensitive and appropriately adapted to the local dialect and comprehension level.

#### Sample and recruitment

Participant recruitment took place between 2018 and 2021, with a 13-month interruption due to the COVID-19 pandemic. Participants were identified through partnership with scientific experts conducting dengue research in Peru and Ecuador. To be eligible, patients had to be 1–65 years of age (inclusive), have a laboratory-confirmed diagnosis of dengue within the last 30 days, been asymptomatic for at least 1 day, and only observed/treated for dengue as an outpatient. Participants were recruited across three age-groups: adults aged 18 years or older, adolescents/older children aged 8–17 years accompanied by their caregiver, and caregivers of younger children aged 1–7 years who had experienced dengue illness. A target of 20 interviews in each age group was expected to be sufficient for achieving ‘concept saturation’ (a point at which no new concepts are likely to emerge with further interviews) [25, 26]. Demographic and clinical information were also collected. All participants were compensated for participation as regionally appropriate.

#### Ethics

The study was approved and overseen by the U.S. Naval Medical Research Unit No. 6 Institutional Review Board (IRB) in Peru (NAMRU6.2014.0028) and by central SUNY Upstate IRB (417710) and Luis Vernaza Hospital (HLV-CEISH-2020-005) in Ecuador, in compliance with all applicable federal regulations governing the protection of human subjects. All participants provided written informed consent and/or assent (for children aged 8 years and above only) prior to study-related activities.

#### Interview procedure

Interviews were 60-min and conducted face-to-face or via telephone in the participant’s native language by site investigators trained in qualitative interviewing, using a semi-structured interview guide. Older children and adolescents (aged 8–17 years) were interviewed with their caregiver; however, interviewers were encouraged to engage primarily with the child/adolescent.

The CE section of the interviews used broad, open-ended questions to facilitate spontaneous, unbiased elicitation of concepts regarding the patient experience of dengue. Focused questions were used if concepts of interest had not emerged or been fully explored.

For the CD section, participants were asked to complete the DENV-DD either on paper or electronically on a device using a ‘think aloud’ approach [27] and asked to share their thoughts as they read each instruction/item and selected each response. During both round one and round two interviews, the PRO was debriefed with adult patients aged 18–65 and patients who were older children/adolescents aged 8–17 (with their caregiver). The ObsRO was debriefed with caregivers of patients aged 1–7. Participants were asked detailed questions about their interpretation and understanding of instructions/item wording and the recall period, relevance of concepts, and appropriateness of response options.

#### Qualitative analysis

All interviews were audio-recorded, transcribed verbatim, and translated to English, with identifiable information redacted. The CE section of the transcripts was thematically analyzed using Atlas.ti software [28]. Participant quotes pertaining to signs/symptoms and impacts of dengue were assigned corresponding concept codes in accordance with an agreed coding list.

Concept saturation analysis was conducted at the total sample level and age-group level, examining symptom concepts only, given the focus of the study was on symptom assessment. Transcripts were chronologically grouped into equal sets with findings from each set iteratively compared. Saturation was deemed to have been achieved if no new spontaneously reported concepts emerged in the final set.

For the CD section of the transcripts, dichotomous codes were assigned to each item, instruction, response option(s), and recall period to indicate whether it was understood, relevant, and/or appropriate, and why. Further codes captured any suggested changes.

In some instances, caregivers of younger children were incorrectly debriefed using the PRO rather than the ObsRO. To mitigate for this, understanding and relevance was extrapolated from PRO items that were similarly worded and conceptually equivalent to ObsRO items (e.g. PRO: “I felt tired”, ObsRO: “My child felt tired); relevance was further informed by responses given during CE.

### Quantitative assessment

An independent, small-scale quantitative assessment was conducted in the context of a DHIM (NCT04298138, [29, 30]) to generate preliminary evidence of feasibility of DENV-DD completion throughout illness, instrument performance and early insight into the trajectory of signs/symptoms over the course of illness. The study was funded by the United States Army and sponsored and executed by the State University of New York, Upstate Medical University (ethical approval obtained from a centralized US IRB, WCG IRB: 20193154).

#### Quantitative assessment study procedures

Participants were recruited from non-dengue endemic areas in the Northeast US between August and December 2022 as part of the DHIM. Healthy volunteers were experimentally infected with a single dose of dengue virus serotype 3 (DENV-3); 0.5 ml of 1.4 × 10^3^ pfu/ml. Study investigators monitored the development of dengue-associated signs/symptoms and tested participants for dengue via polymerase chain reaction (PCR) tests which were conducted at study visits scheduled throughout the 28-day study period.

On day one of experimental infection, participants were provided with printed paper copies of the US-English paper version of the DENV-DD PRO (modified following round one qualitative interviews) and instructed to complete one every day for 28 days. Participant completed DENV-DD daily entries were reviewed by the study investigators at each study visit.

#### Quantitative analysis

Data from the DENV-DD paper diary entries was electronically scanned and then manually entered into a Microsoft Excel database. To ensure accuracy of data entry, 20% of the data was inputted by two individuals and then compared. Any discrepancies were checked against the scanned version of the diary.

Descriptive analyses were conducted on the raw item-level diary ratings to provide early insight into item performance. Compliance with completing the diary every day for 28 days (throughout an episode of dengue) was assessed by looking at the level of missing data. The item-level response distributions were used to assess the appropriateness of the response options. Alongside the qualitative interview findings, this data was used to support instrument modifications. The data also provided additional insight into the progression of symptoms over the course of illness, including: the timing, duration, and intensity of symptoms. All analyses were pre-specified and detailed in the analysis plan prior to data collection.

## Results^1^

### Qualitative study sample demographic and clinical characteristics

A total of 48 participants were interviewed: 20 adults (round 1: n = 13, round 2: n = 7), 20 adolescents/older children with their caregiver (round 1: n = 12, round 2: n = 8), and eight caregivers of children (round 1: n = 6, round 2: n = 2). Participants were recruited from Peru (n = 39/48, 81%) and Ecuador (n = 9/48, 19%).

Patients ranged in age from 1 to 53 years and were generally evenly split between female (n = 25/48, 52%) and male (n = 23/48, 48%), although there was a larger proportion of male patients from Ecuador (n = 7/9, 78%) compared to Peru (n = 16/39, 41%) (Table 1). Within each age group, there was representation of participants across different school grades/levels of education. From those reported, most patients had DENV-1 (n = 30/33, 91%), and one patient reported having had dengue previously. Only patients from Peru were debriefed on the electronic device.

**Table 1:**
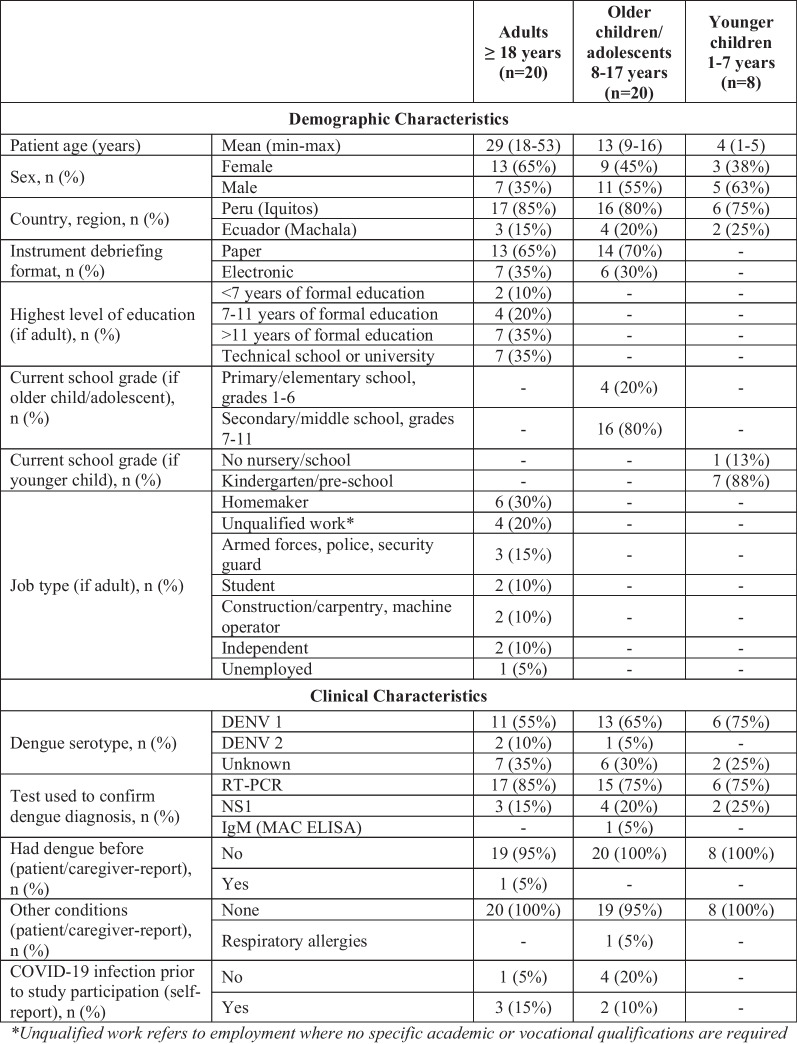
Demographic and clinical characteristics of patients with dengue by age group

*DENV 1/2* dengue virus stereotype 1/2, *RT-PCR* reverse transcription polymerase chain reaction, *NS1* non-structural protein, *IgM (MAC ELISA* immunoglobulin M antibody-capture enzyme-linked immunosorbent assay

Caregivers ranged in age from 22 to 52 years, and most were the child’s mother (n = 25/28, 89%) (Table 2).

**Table 2:**
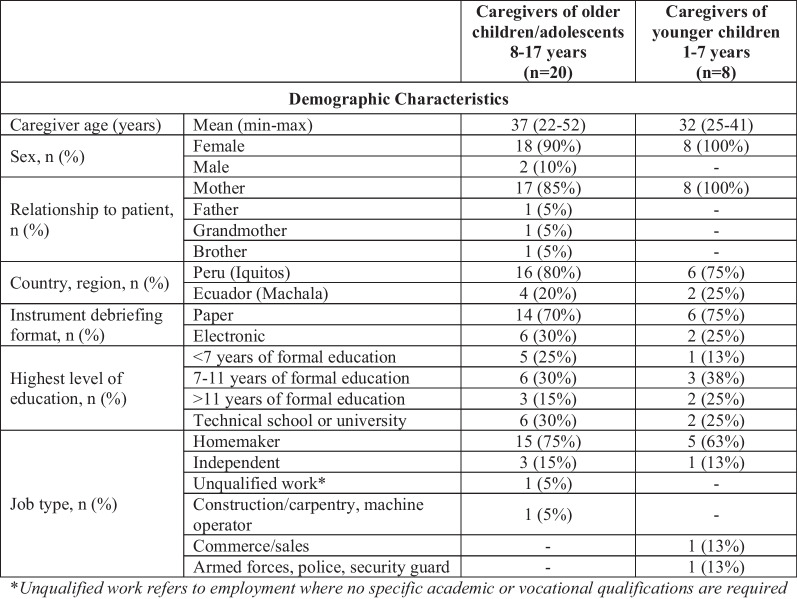
Demographic and clinical characteristics of caregivers by patient age group

### Concept elicitation results

#### Participant experience of dengue

**Fig. 3:**
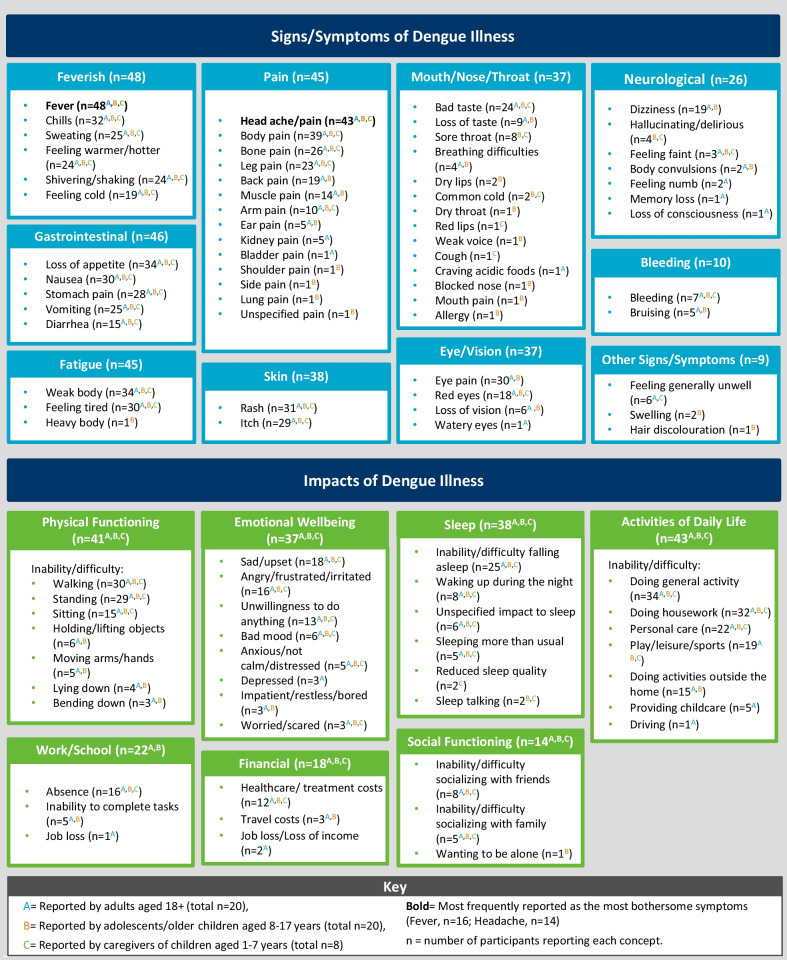
Summary of the concepts relating to the dengue illness experience as reported by participants, either spontaneously or when probed, during qualitative interview

The findings from both rounds of CE interviews are summarized in Fig. 3.

**Fig. 4:**
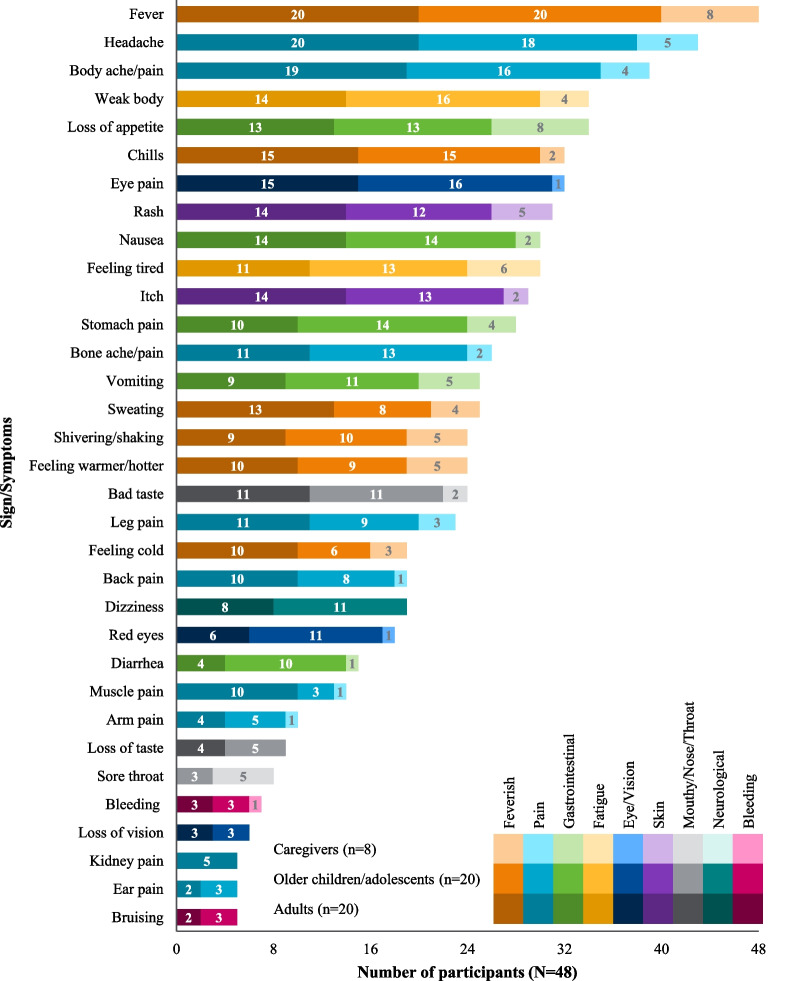
Frequency of signs/symptoms reported by participant type, either spontaneously or when probed. Color denotes high-level sign/symptom category. Color value (lightness) denotes age group

**Fig. 5:**
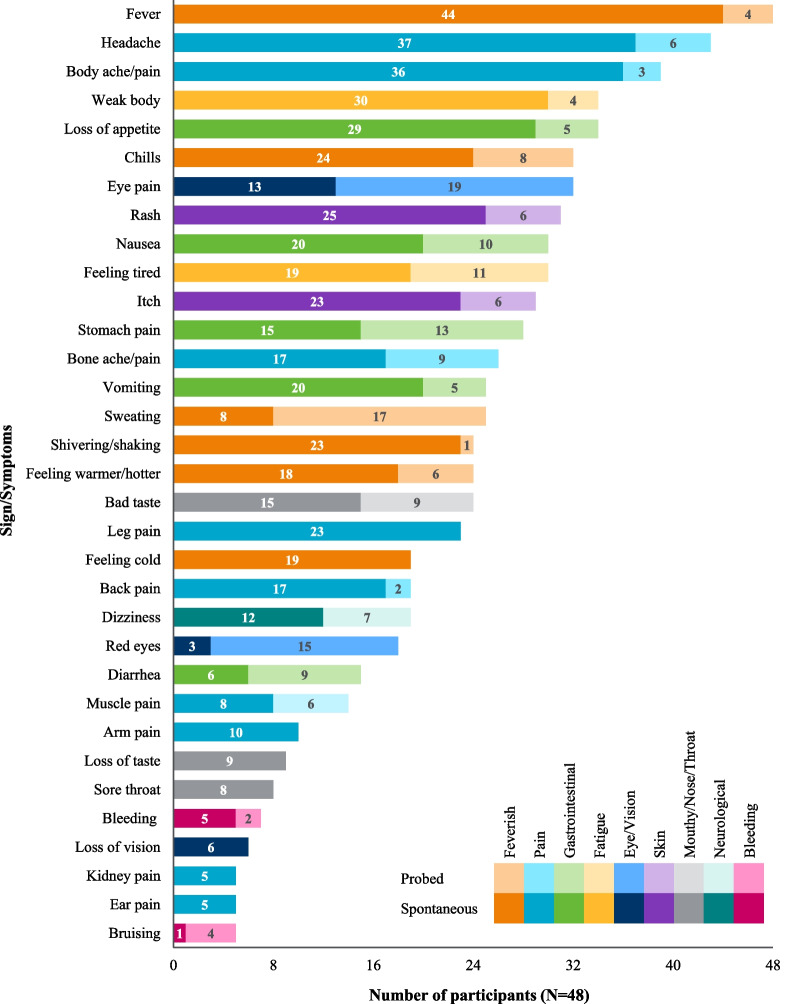
Frequency of signs/symptoms reported across all qualitative interviews, either spontaneously or when probed. Color denotes high-level sign/symptom category. Color value (lightness) denotes spontaneous/probed

Participants reported a total of 59 signs/symptoms, which broadly extended across 10 categories: feverish, gastrointestinal, pain, fatigue, skin, mouth/nose/throat, eye/vision, neurological, bleeding, and other (see Table 3 for example quotes; Spanish translations: Additional file 1: Table S1). At the symptom-level, fever (n = 48/48, 100%), headache (n = 43/48, 90%), body ache/pain (n = 39/48, 81%), loss of appetite (n = 34/48, 71%), and body weakness (n = 34/48, 71%) were most frequently reported (see Fig. 4 for a breakdown of signs/symptoms reported by participant type and Fig. 5 for a breakdown of signs/symptoms reported either spontaneously or when probed).

**Table 3:** Key sign/symptom categories with example participant quotes

Sign/symptom categories	Participant quote
Feverish (n = 48)	“**Participant (adult):** And after a while of…of having the chills, I got hit with a…a fever, a really high fever.” (chills/fever; 31 years old female from Iquitos, Peru) “**Participant (caregiver):** I would feel him and he would be really hot, with a fever.” (feeling warmer/hotter/fever; caregiver of a 4 year old male from Iquitos, Peru)
Gastrointestinal (n = 46)	“**Participant (adolescent):** I was disgusted, I didn´t want to eat, even just seeing it I felt disgusted.” (loss of appetite; 13 year old male from Iquitos, Peru) “**Participant (caregiver):** My son told me that his stomach hurt.” (tummy pain; caregiver of a 4 year old male from Iquitos, Peru)
Pain (n = 45)	“**Participant (child):** I felt like my head was going to explode right then.” (headache; 12 year old female from Machala, Ecuador) “**Participant (caregiver):** The pain—the body aches, yes, there were moments. There were a few moments he’d lay down, feel better, that is. He could move, walk. Then it would flare up again.” (body pain; caregiver of a 4 year old male from Iquitos, Peru)
Fatigue (n = 45)	“**Participant (adult):** I could do things, but very slowly, it was difficult for me, because I was very tired” (feeling tired; 21 year old male from Machala, Ecuador) “**Participant (adolescent):** I felt weak- I didn’t have any strength.” (weak body; 16 year old female from Iquitos, Peru)
Skin (n = 38)	“**Participant (adult):** I had a rash over my whole body, my whole body was completely red, red and swollen.” (rash; 27 year old female from Iquitos, Peru) “**Participant (adolescent):** When I wanted to sleep, that’s when I started to feel itchy. Then I moved my leg and the itchiness started to get worse and worse…” (itch; 13 year old male from Iquitos, Peru)
Eye/vision (n = 37)	“**Participant (adult):** I would feel the pressure here in this area of the eyebrows. I would bend over and I would feel a pressure coming down.” (eye pain; 23 year old female from Iquitos, Peru) “**Participant (adolescent):** They were turning red like vampire.” (red eyes; 15 year old male from Iquitos, Peru)
Mouth/nose/throat (n = 37)	“**Participant (adolescent):** I couldn’t eat. I had a sour taste in my mouth.” (bad taste; 15 year old female from Iquitos, Peru) “**Participant (caregiver):** Her throat was sore. She could eat, she would eat, and she would put her hand on where she was sore.” (sore throat; caregiver of a 5 year old female from Iquitos, Peru)
Neurological (n = 26)	“**Participant (adult):** Dizzy, yes … you feel like you´re drunk, like I was about to fall over.” (dizziness; 38 year old female from Iquitos, Peru) “**Participant (child):** I ju… just felt dizzy when I walked.” (dizziness; 9 year old male from Machala, Ecuador)
Bleeding (n = 10)	“**Participant (caregiver):** [he] did start bleeding and started spitting blood.” (bleeding; caregiver of a 9 year old male from Iquitos, Peru) “**Interviewer:** Did you have bruises at any point? **Participant (adolescent)***:* I had some spots on my arms … Brown.” (bruising; 16 year old female from Iquitos, Peru)

*n* number of participants reporting each sign/symptom concept

Total symptom duration ranged from 3 to 15 days, with 5 days (n = 7/35, 20%) and 7 days (n = 8/35, 23%) most frequently reported. Fever (n = 16/37, 43%) and headache (n = 14/37, 38%) were reported to be the most bothersome symptoms. Findings indicated symptom occurrence was transient across an individual’s illness, and frequency and intensity of individual symptoms was highly variable across individuals.

All participants reported how dengue impacted HRQoL. Impacts to activities of daily living (n = 43/48, 89%), physical functioning (n = 41/48, 85%), sleep (n = 38/48, 79%), emotional wellbeing (n = 37/48, 77%), work/school (n = 22/48, 46%), finances (n = 18/48, 38%), and social functioning (n = 14/48, 29%) were mentioned.

Almost all participants (n = 47/48, 98%) discussed using supportive treatments to alleviate dengue symptoms, with paracetamol and increased fluid intake (n = 33/47, 70%, each) most frequently mentioned.

#### Concept saturation

Concept saturation was achieved for the total sample, with no relevant concepts emerging in the final set of interviews (grouped into four sets of 12 interviews).

At the age-group level, concept saturation was achieved for both the adult and older children/adolescent samples (both independently grouped into four sets of five interviews). For caregivers of younger children (grouped into four sets of two interviews), three symptom concepts emerged in the final set of interviews, including muscle and back pain, which are core symptoms of dengue [10], suggesting further interviews may have elicited additional concepts in this age group.

### Cognitive debriefing of the DENV-DD

#### DENV-DD PRO

In round one, adults (n = 13) and older children/adolescents (n = 12) completed and debriefed the 22-item DENV-DD PRO. All 22 items were understood by ≥ 80% participants and most items (18/22) were considered relevant to ≥ 50% participants. Items assessing vomiting, diarrhea, bruising, and taking temperature were considered relevant to < 50% of participants. Most instructions were understood; some participants had difficulty understanding the recall period (n = 7/23, 30%). All participants demonstrated an understanding of the response options (n = 25/25, 100%).

Based on round one findings and input from the SC, modifications were made to the DENV-DD PRO item wording and instructions to enhance understanding and relevance. Five items were added to the PRO to assess concepts reported during the interviews not captured by the DENV-DD (weak body, back pain, bad taste, bleeding, dizziness), and one item assessing any treatments taken was included.

In round two, adults (n = 7) and older children/adolescents (n = 8) completed and debriefed the updated 28-item DENV-DD PRO. Findings indicated most items (27/28) were understood by ≥ 80% participants. Most items (24/28) were also considered relevant to ≥ 50% participants. Diarrhea, bruising, bleeding, and taking temperature were considered relevant to < 50% of participants. Most instructions were understood; some still had difficulty understanding the re-worded recall period (n = 2/6, 33%). Nearly all participants demonstrated understanding of the response options (n = 14/15, 93%).

Additional modifications were made to the DENV-DD PRO based on round two findings and input from the SC, including further updates to the instructions to clarify the recall period. Two items were added to assess concepts reported during the interviews: sore throat and leg pain.

#### DENV-DD ObsRO

In round one, six caregivers of younger children were debriefed on the DENV-DD. Four were debriefed on the 21-item ObsRO and two debriefed on the PRO. Most items (16/21) were well-understood by ≥ 80% participants and most (17/21) were considered relevant to ≥ 50% participants. Items assessing diarrhea, scratching, bruising, and red eyes were considered relevant to < 50% of participants. The instructions were broadly understood by all participants asked; most had difficulty understanding the recall period (n = 4/6, 67%). All participants asked demonstrated an understanding of the response options (n = 5/5, 100%).

Following round one, modifications to item and instruction wording were made to the DENV-DD ObsRO, mostly to align with changes made to the DENV-DD PRO. Six items were added based on concepts reported in CE: weak body, bad taste, bleeding, dizziness, fever, sore throat, and one item was added to assess any supportive treatments taken.

In round two, two caregivers of younger children were interviewed, both of whom were debriefed on the PRO. Most items (25/28) were understood by both participants; the remaining three items were not debriefed with either participant due to interview time constraints/no comparable item being included within the PRO. Most items (18/28) were considered relevant to at least one participant. Nausea, diarrhea, bad taste, bruising, bleeding, dizziness, and red eyes were not relevant to participants who were asked. The instructions were broadly understood by both participants; one had difficulty understanding the re-worded recall period. The one participant asked about the response options demonstrated an understanding.

Following round two, modifications were made to the DENV-DD ObsRO instruction wording to align with changes to the DENV-DD PRO.

### Hypothesized conceptual framework

**Fig. 6:**
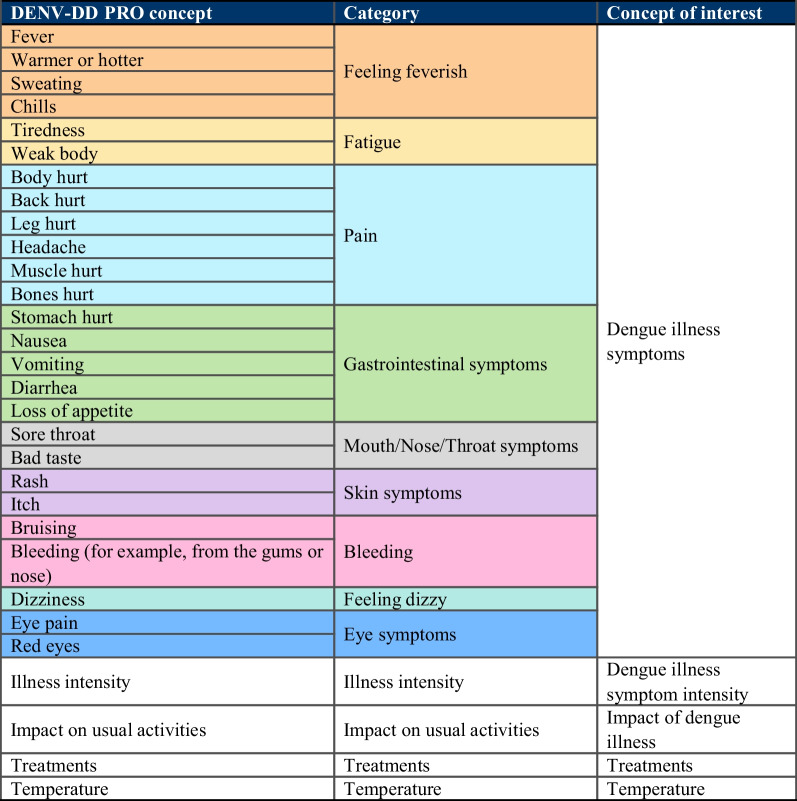
Hypothesized conceptual framework of the DENV-DD PRO. *Note:* Shaded groups denote conceptual categories of symptom items. Items which are unshaded do not fall into specific symptom concepts

**Fig. 7:**
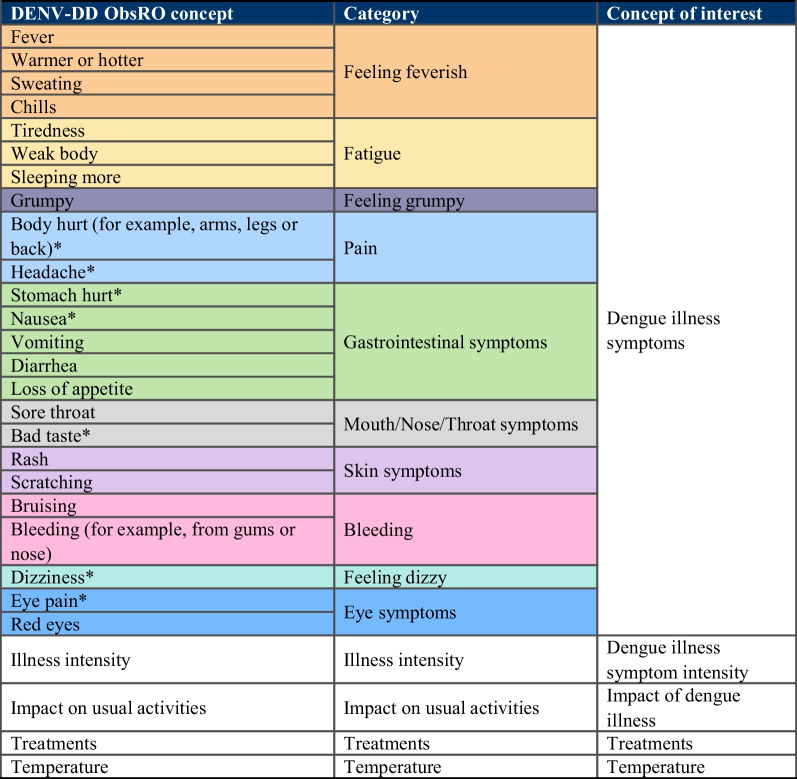
Hypothesized conceptual framework of the DENV-DD ObsRO. *Note*: Shaded groups denote conceptual categories of symptom items. Items which are unshaded do not fall into specific symptom concepts. *ObsRO concept may not be directly observable and is unlikely to be articulated clearly to caregivers from very young children. These concepts include a ‘I don’t know’ response option for caregivers within the DENV-DD ObsRO

Both the DENV-DD PRO (Fig. 6) and ObsRO (Fig. 7) assess the nine core dengue sign/symptom categories identified from CE interviews, original DII-RC and published literature [2]. The PRO contains four additional pain items (back, leg, muscle, and bone pain), while the ObsRO captures an additional concept of ‘feeling grumpy’. Individual items capturing illness intensity, impact of illness, treatments taken, and temperature are also included in DENV-DD versions. These individual items will likely be scored separately to the DENV-DD total score, with findings from future psychometric validation evaluation informing final item inclusion, scoring domains and algorithms. The global illness intensity and impact of illness items will also be used as anchor measures to support psychometric evaluation of the DENV-DD.

### Comparison between age groups

Experiences of dengue illness across the whole sample were generally very similar. However, adults and older children/adolescents were able to provide highly detailed descriptions of their experience, whereas caregivers of younger children were only able to communicate what they had observed or been told by their child (e.g. fewer caregivers reported their child experienced pain and eye/vision-related symptoms). The similarity in reporting by adults and older children/adolescents during CE, and the fact that no major differences in item understanding and concept relevance were identified between these age-groups during CD, supports use of the PRO in adults and older children/adolescents. The difference in reporting by caregivers supports the need for an ObsRO for assessment of younger children [17].

### Quantitative assessment sample demographic and clinical characteristics

Nine participants were recruited as part of the DHIM study. Starting on Day 2–4 post-inoculation, all individuals were clinically diagnosed with dengue illness.

Participants ranged in age from 22 to 40 years and were generally evenly split between female (n = 5/9, 56%) and male (n = 4/9, 44%). All participants had a high school diploma or higher. Two thirds of participants (n = 6/9, 67%) were hospitalized for 2–5 days (mean: 3.2 days) (Table 4).

**Table 4:**
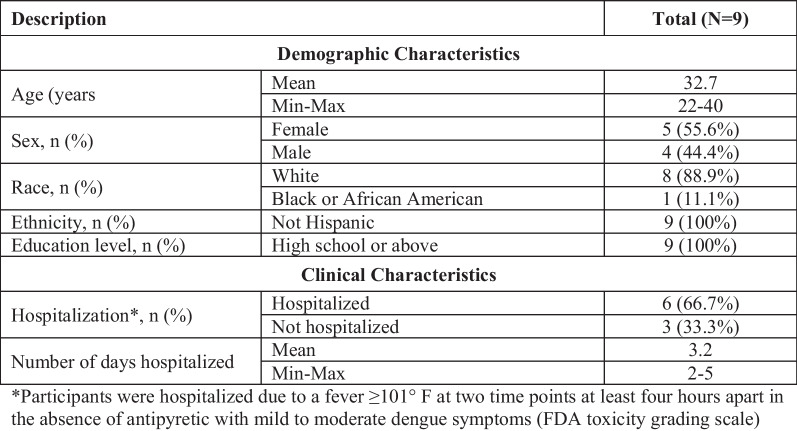
Sample demographic and clinical characteristics for the quantitative sample

### Quantitative assessment results

DENV-DD form completion rate was high (89.7%), with three participants completing the DENV-DD every day for 28 days (Additional file 1: Figure S1). Item-level completion rate, for completed forms, was very high at 99.5% (Additional file 1: Figure S2), demonstrating feasibility of completing the entire DENV-DD every day.

**Fig. 8:**
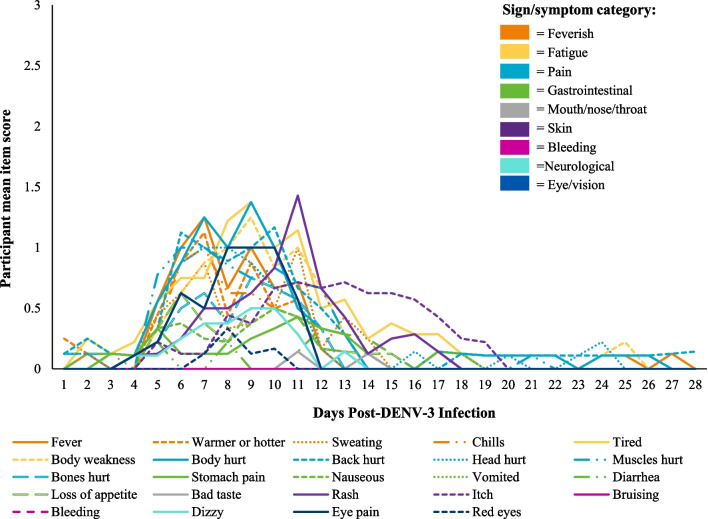
Sample mean item rating for the 24 symptom items across 28 days of diary completion. A higher rating indicates greater symptom intensity. For each item, on each day post DENV-3 infection, participant mean rating was calculated as the sum of all participant responses divided by the number of participant respondents. Missing data was not included in the mean rating calculation

Participants endorsed the full range of response options on the DENV-DD during the 28-day diary completion period with no evidence of ceiling effects. The intensity of individual symptom reporting for each participant peaked between Days 6–13 (Fig. 8). Feverish, pain, and fatigue symptoms were experienced earliest following inoculation, followed by skin, mouth/nose/throat and eye/vision symptoms. Average symptom duration was 12 days. Back pain and tiredness were experienced for the longest duration (20–24 days), while bad taste and red eyes were experienced for the shortest duration (2–4 days). Of the 24 symptom items assessed in the DENV-DD, most were experienced by at least one participant (21/24). Vomiting, bleeding and bruising were not experienced.

## Discussion

This study added to the understanding of the outpatient dengue illness experience [31, 32]. Consistent with previous literature [10], fever, headache, musculoskeletal pain, fatigue, rash, and nausea/vomiting were identified as core signs/symptoms of dengue. Less commonly reported signs/symptoms in the literature (i.e. bad taste and sore throat) were also identified, both of which were reported as important to the dengue experience in a recent community-based perspective study [32]. Some signs/symptoms (e.g. craving acidic foods, allergy-like symptoms, watery eyes, and loss of consciousness) were each reported by only one participant. None of these signs/symptoms are supported in the literature as being related to dengue [10, 32], thus may be attributable to a co-morbid condition(s).

Findings also support the previous literature [33, 34] that dengue signs/symptoms fluctuate throughout the course of illness and this experience often varies between individuals with the disease. This variability in illness trajectory and symptom presentation/intensity supports the need for instruments to capture the full dengue patient experience.

### Content validity of the DENV-DD

The current study provides evidence to support the content validity of the DENV-DD. Most participants understood the DENV-DD PRO and ObsRO instructions, items, and response options as intended and indicated most concepts assessed by the DENV-DD were relevant to their (or their child’s) dengue experience. Minor changes to instruction/item wording were made across rounds of interviews based on participant and SC feedback to enhance understanding and relevance to the dengue population. Items assessing weak body, bad taste, sore throat, bleeding, dizziness, and treatments taken were added to the DENV-DD PRO and ObsRO; an item assessing fever was added to the ObsRO (already included in the PRO); and items assessing back pain and leg pain were added to the PRO. While relevance was consistently reported to be low for diarrhea, bruising, and bleeding items, input from the scientific committee recommended retaining the items as they are established (albeit less common) symptoms of dengue illness [32], with bruising and bleeding potentially indicating progression to clinically severe dengue [10].

Given the nature of dengue illness (symptoms fluctuating in intensity over the course of illness), the response scale was deemed suitable for capturing the gradation in symptom intensity. Some participants had difficulty explaining what the 24-h recall period meant. Difficulty in understanding was likely exacerbated due to participants no longer having dengue at the time of the interview and being asked to reflect on their experience over the time of their dengue illness. From an instrument development perspective, a 24-h recall period was deemed most appropriate to minimize recall bias and to capture daily symptom fluctuation [14, 35]. Results of the quantitative assessment conducted with symptomatic participants with recent dengue onset provided evidence of the feasibility of daily DENV-DD completion, indicating that any critical issues with recall period were resolved when completing the diary in real-time.

This led to the 30-item DENV-DD PRO and 28-item DENV-DD ObsRO.

### Study considerations

In Peru and Ecuador, dengue is an illness principally experienced by low to middle socio-economic populations [36, 37]. Local research investigators and healthy individuals were engaged to ensure cultural appropriateness and understandability of the Spanish translations across all ages and literacy levels. A broader definition of ‘caregiver’ (e.g. siblings, grandparents) was applied to better reflect the family dynamics in South America where children often live in multi-generational households or with individuals other than their parents. Interviews were conducted mostly in-person to improve discourse and rapport between interviewers and participants and to mitigate issues with poor internet connectivity.

Although a range of age groups were included in this study, the number of participants recruited in the caregiver sample representing younger children who had experienced dengue was quite small, thus concept saturation for this sample was not achieved. This may reflect a lower prevalence of symptomatic dengue in younger children in South America [38], but also is likely due to a 13-month interruption to data collection during the COVID-19 pandemic. Half of the caregiver sample was also inadvertently debriefed on the DENV-DD PRO instead of the ObsRO, however data for PRO items that were conceptually equivalent to ObsRO items, and CE findings, were used to extrapolate data on understanding and relevance. Further interviews with caregivers could be conducted to confirm content validity of the ObsRO.

The qualitative interview findings should not be considered an estimate of symptom prevalence. Most participants were identified through clinics based on the presence of ‘classic’ dengue symptoms (e.g. fever). Hospitalized patients were excluded, resulting in clinically severe dengue not being captured, despite symptoms of bruising and bleeding which are indicative of a more severe dengue symptomology being discussed. Symptom prevalence was not an objective of this study but could be examined using the DENV-DD in future research studies. While the items more indicative of severe dengue were not tested in a population clinically diagnosed with severe dengue, the study sample was selected from a population that is at risk for severe dengue illness (i.e. dengue endemic populations). This study sample would be sufficient to demonstrate item understanding. The ongoing psychometric validation study will evaluate the performance of items, in a sample with differentiating disease intensities, allowing the opportunity for items demonstrating low relevance to be removed at this stage.

Further, although the quantitative assessment provided early evidence of the feasibility of DENV-DD completion, instrument performance and insight into the trajectory of the signs/symptoms of dengue illness, due to the small sample size robust conclusions cannot be drawn from the data. Additionally, as participants in this research study were healthy trial volunteers who were experimentally infected, the typical patient experience may not have been captured. Further observational work is currently underway in Southeast Asia to provide more robust real-world data to support use of the DENV-DD in outpatient populations.

### Next steps

To evaluate item performance, psychometric properties and develop a scoring algorithm and score interpretation guidelines, an observational study is underway in Southeast Asia where DENV-DD responses will be collected daily from patients and caregivers of patients with dengue illness. Items will be reviewed to minimise redundancy and to optimize the balance between conceptual coverage and burden of completion. The cultural relevance of the DENV-DD in new populations will also be assessed.

## Conclusions

The DENV-DD was developed with qualitative input from patients/caregivers of patients who had recently experienced dengue, in accordance with regulatory guidance [14–16]. The DENV-DD PRO and ObsRO versions have documented evidence of face and content validity for the assessment of signs/symptoms of dengue illness for use with outpatient children, adolescents, and adults. Observational study data will be used to evaluate the psychometric measurement properties of the DENV-DD to support its use to characterize dengue burden and assess therapeutic value in future clinical research studies and real-world applications.

### Supplementary Information

**Additional file 1**. **Table S1.** Key sign/symptom categories, where n = number of participants reporting each sign/symptom concept, with example participant quotes in Spanish. **Figure S1.** Form-level completion of the 28-item DENV-DD PRO. **Figure S2.** Item-level completion of the 28-item DENV-DD PRO.

## Data Availability

All the relevant data has been reported in the manuscript and supplementary files. The datasets generated during and/or analyzed during the current study are not publicly available due to participants consenting to data being published in anonymized form and full interview recordings/transcripts only being available to the project team responsible for conducting the research.

## Abbreviations

CD: Cognitive debriefing
CE: Concept elicitation
COA: Clinical outcome assessment
DENV-3: Dengue virus serotype 3
DENV-DD: Dengue Virus Daily Diary
DII-RC: Dengue Illness Index Report Card
DHIM: Dengue human infection model
HRQoL: Health related quality of life
IRB: Institutional Review Board
ObsRO: Observer-reported outcome
PCR: Polymerase chain reaction
PRO: Patient-reported outcome
WHO: World Health Organization

## Footnotes

1 Throughout the results section, percentages are reported out of the total number of participants who were questioned/debriefed on the specific concept/topic.
