# Supplementary material for: Measuring dengue illness intensity: Development and content validity of the dengue virus daily diary (DENV-DD)
Source: J Patient Rep Outcomes. 2023 Aug 23;7:84. doi: 10.1186/s41687-023-00624-5 (PMC10447358; doi:10.1186/s41687-023-00624-5)
Supplement: Supplementary file 1 — Additional file 1. Table S1. Key sign/symptom categories, where n = number of participants reporting each sign/symptom concept, with example participant quotes in Spanish. Figure S1. Form-level completion of the 28-item DENV-DD PRO. Figure S2. Item-level completion of the 28-item DENV-DD PRO. [file 41687_2023_624_MOESM1_ESM.docx]

**Supplementary Material**

# **1.0 Concept elicitation results (Spanish Quotes)**

| *Table S1. Key sign/symptom categories, where n = number of participants reporting each sign/symptom concept, with example participant quotes in Spanish.* | |
| --- | --- |
| **Sign/symptom categories** | **Participant quote (original Spanish)** |
| Feverish (n=48) | *“****Participante:*** *Y, al rato que, que estaba con escalofrío, me agarró la, la fiebre, fiebre bien alta”* (chills/fever; 31 year old female from Iquitos, Peru)  *“****Participante:*** *Y, ya me iba a tocarlo, y bien caliente, con fiebrecita”* (feeling warmer/hotter/fever; caregiver of a 4 year old male from Iquitos, Peru)*.* |
| Gastrointestinal (n=46) | *“****Participante (son):*** *Me dio mucho asco, no quería comer, al verlo nada mas sentía asco”* (loss of appetite; 13 year old male from Iquitos, Peru)  *“****Participante:*** *Mi hijo me dijo que le dolía el estómago”* (tummy pain; caregiver of a 4 year old male from Iquitos, Peru) |
| Pain (n=45) | *“****Participante:*** *Me sentía que la cabeza me iba a explotar ahí.”* (headache; 12 year old female from Machala, Ecuador)  *“****Participante:*** *El dolor del – de cuerpo, sí había momentos, había un, un rato que se acostaba más tranquilo, dijo. Podía ya moverse, andar. Después cuando ya le atacaba otra vez, se tenía que, que echarse”* (body pain; caregiver of a 4 year old male from Iquitos, Peru) |
| Fatigue (n=45) | *“****Participante:*** *…que podía hacer esas, pero muy lento, me costaba mucho, porque estaba muy cansado”* (feeling tired; 21 year old male from Machala, Ecuador)  *“****Participante:*** *Si, me sentía débil- no tenia fuerzas”* (weak body; 16 year old female from Iquitos, Peru) |
| Skin (n=38) | *“****Participante:*** *Tuve ronchas en todo mi cuerpo, todo mi cuerpo se me había hecho bien rojo. Rojo e hinchado, todo mi cuerpo estaba hinchado, hasta mis pies”* (rash; 27 year old female from Iquitos, Peru)  *“****Participante:*** *…quise dormir sentí una pequeña comezón, y luego moví la pierna y la comezón empezó a sentirse más fuerte y mas fuerte…”* (itch; 13 year old male from Iquitos, Peru) |
| Eye/vision (n=37) | *“****Participante:*** *…sentía la presión acá en esta parte de las cejas yo me agachaba así y sentía una presión que se venía hacia abajo”* (eye pain; 23 year old female from Iquitos, Peru)  *“****Participante:*** *Se estaban poniendo rojos como un vampiro”* (red eyes; 15 year old male from Iquitos, Peru) |
| Mouth/nose/throat (n=37) | *“****Participante:*** *No, no podía. Sentía amargo en mi boca”* (bad taste; 15 year old female from Iquitos, Peru)  *“****Participante:*** *le dolía la garganta. No podía comer; comía, y se agarraba donde le dolía”* (sore throat; caregiver of a 5 year old female from Iquitos, Peru) |
| Neurological (n=26) | *“****Participante:*** *Mareada si …pareces borracha, que me voy a caer”* (dizziness; 38 year old female from Iquitos, Peru)  *“****Participante:*** *No. So… solo me mareaba cuando caminaba”* (dizziness; 9 year old male from Machala, Ecuador) |
| Bleeding (n=10) | *“****Participante:*** *si comenzó a sangrar y comenzó a escupir sangre”* (bleeding; caregiver of a 9 year old male from Iquitos, Peru)  *“****Interviewer: ¿****Tenías moretones en algún momento?*  ***Participante:*** *En mis brazos me salieron unas manchas…Marrones”* (bruising; 16 year old female from Iquitos, Peru) |

# **2.0 Quantitative assessment – Form-level missing data**


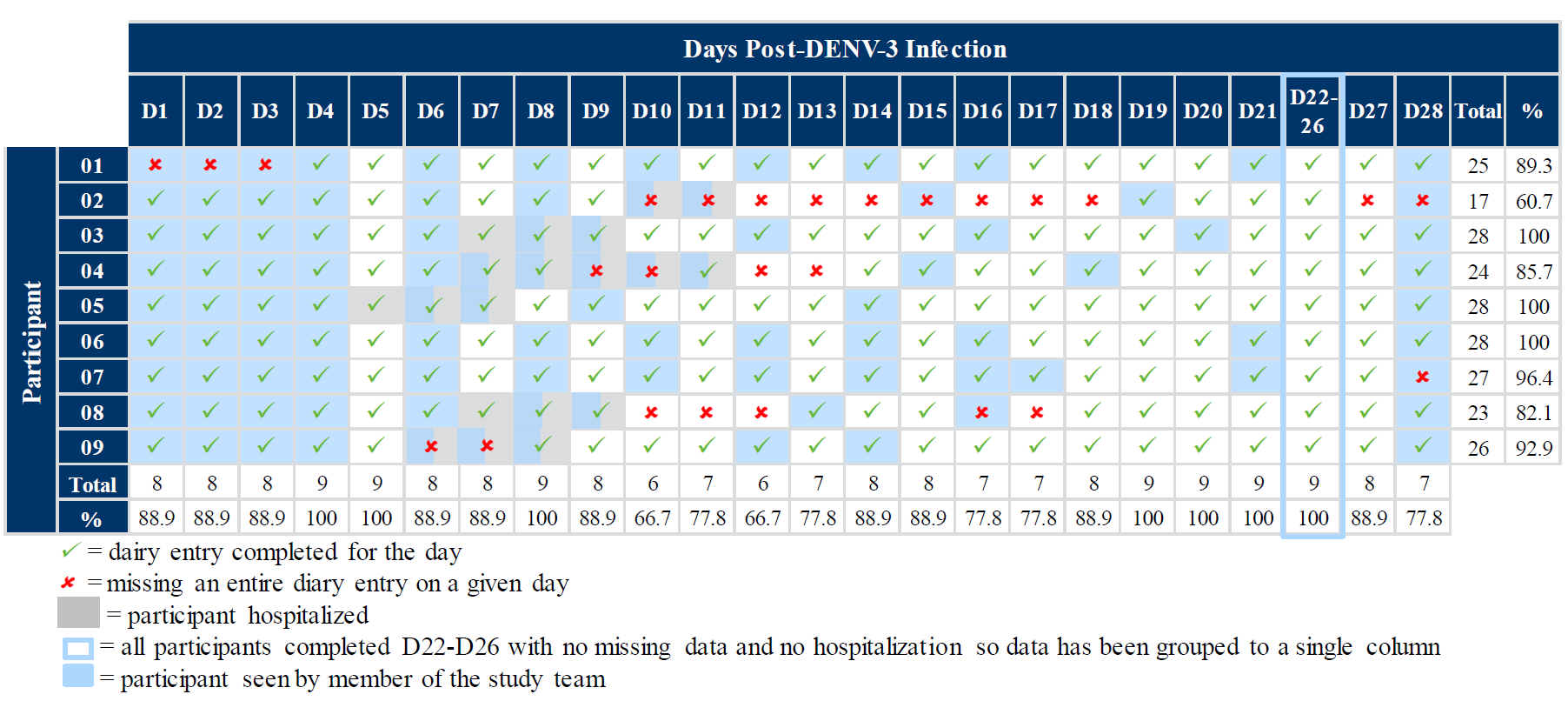


Figure S1. Form-level completion of the 28-item DENV-DD PRO.

# **3.0 Quantitative assessment – Item-level missing data**


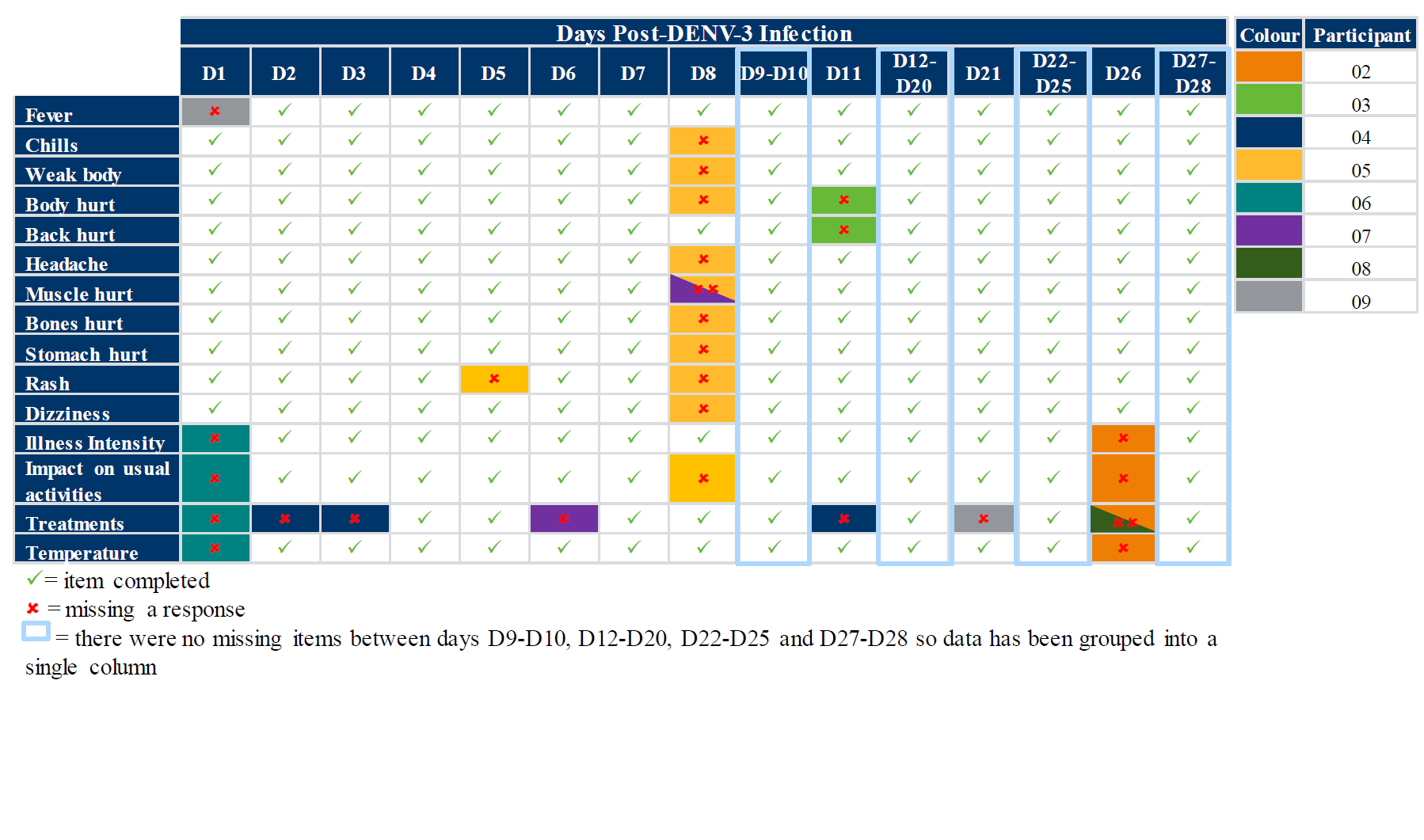


Figure S2. Item-level completion of the 28-item DENV-DD PRO.
